# Red Blood Cell Partitioning Using a Microfluidic Channel with Ladder Structure

**DOI:** 10.3390/mi14071421

**Published:** 2023-07-14

**Authors:** Toru Hyakutake, Yuya Tsutsumi, Yohei Miyoshi, Manabu Yasui, Tomoki Mizuno, Mizuki Tateno

**Affiliations:** 1 Faculty of Engineering, Yokohama National University, 79-5 Hodogaya, Yokohama 240-8501, Japan; 2 Graduate School of Engineering Science, Yokohama National University, 79-5 Hodogaya, Yokohama 240-8501, Japan; tsutsumi-yuya-mk@ynu.jp (Y.T.); miyoshi-youhei-bf@ynu.jp (Y.M.); mizuno-tomoki-xd@ynu.jp (T.M.); 3 Kanagawa Institute of Industrial Science and Technology, 705-1 Shimoimaizumi, Ebina 243-0435, Japan; yasui@kistec.jp; 4 College of Engineering Science, Yokohama National University, 79-5 Hodogaya, Yokohama 240-8501, Japan; tateno-mizuki-zc@ynu.jp

**Keywords:** red blood cell, partitioning, microfluidic channel, capillary network

## Abstract

This study investigated the partitioning characteristics of red blood cells (RBCs) within capillaries, with a specific focus on ladder structures observed near the end of the capillaries. In vitro experiments were conducted using microfluidic channels with a ladder structure model comprising six bifurcating channels that exhibited an anti-parallel flow configuration. The effects of various factors, such as the parent channel width, distance between branches, and hematocrit, on RBC partitioning in bifurcating channels were evaluated. A decrease in the parent channel width resulted in an increase in the heterogeneity in the hematocrit distribution and a bias in the fractional RBC flux. Additionally, variations in the distance between branches affected the RBC distribution, with smaller distances resulting in greater heterogeneity. The bias of the RBC distribution in the microchannel cross section had a major effect on the RBC partitioning characteristics. The influence of hematocrit variations on the RBC distribution was also investigated, with lower hematocrit values leading to a more pronounced bias in the RBC distribution. Overall, this study provides valuable insights into RBC distribution characteristics in capillary networks, contributing to our understanding of the physiological mechanisms of RBC phase separation in the microcirculatory system. These findings have implications for predicting oxygen heterogeneity in tissues and could aid in the study of diseases associated with impaired microcirculation.

## 1. Introduction

Microcirculation plays an essential role in oxygen delivery and carbon dioxide removal throughout the body via red blood cells (RBCs). In particular, the rheological properties of RBCs significantly affect the flow within capillaries because their diameters are comparable. Therefore, investigating the flow characteristics of RBCs through capillaries is necessary to explain the oxygen supply mechanism in microcirculation [1,2,3]. Capillaries are complex and intricate structures that fill the interstitial spaces of tissues via repeated branching. Generally, the hematocrit decreases in low-flow branches and increases in high-flow branches in microvascular bifurcations, a phenomenon known as the Zweifach–Fung effect [4,5]. However, deviations from the Zweifach–Fung effect are also evident under certain conditions. As a result, the distribution characteristics of RBCs in the microvascular network can become complex, making it challenging to predict oxygen heterogeneity in tissues. Moreover, impaired myocardial microcirculation has been recognized as a cause of ischemic heart disease in some patients [6]. Therefore, further research is necessary to deepen our understanding of the mechanisms underlying RBC heterogeneity in the microcirculatory system, particularly in capillary networks.

Pries et al. [7,8] proposed an empirical relationship describing RBC distribution in microvascular bifurcations and examined the influence of plasma separation in vivo, which is useful for predicting the distribution characteristics in a wide range of vessels involved in microcirculation. In vitro studies have been conducted on microfluidic bifurcation channels with channel widths of the order of 10 μm—based on advancements in microfabrication techniques—to simulate capillaries [9,10,11,12]. More recently, in vitro experiments have been conducted using more complex capillary network geometries. Stauber et al. [13] investigated the flow behavior of RBCs in a microfluidic platform that mimicked alveolar capillaries. Kodama et al. [14] investigated the flow rates and hematocrit values in each segment using microchannels that reflected the microvascular networks in rabbit ovaries. Mantegazza et al. [15,16] examined the uneven partitioning characteristics of RBCs using microflow channels with complex honeycomb networks. Merlo et al. [17] investigated the spatial distribution of RBCs, focusing on both square and hexagonal networks, and constructed a modified RBC partitioning model. Hyakutake et al. [18] investigated the RBC distribution in hierarchical networks ranging from 4 to 10 μm and demonstrated that the RBC distribution deviated from Pries’ empirical formula and was influenced by the distance between the bifurcations. Recent studies have also reported deviations from Pries’ empirical formula owing to RBC lingering at the bifurcations [19,20,21]. Furthermore, numerous numerical studies have reported the partitioning of RBCs into microvascular bifurcations [22,23,24,25,26,27,28,29,30,31,32,33].

Although obtaining extensive information on cell distribution is possible with realistic geometries, it can be difficult to quantitatively evaluate the various factors relating to microvascular bifurcation geometries, such as the distance between bifurcations, bifurcation angles, and diameters of the branching vessels. Therefore, classifying capillary network geometries into hierarchical or honeycomb structures, among others, can provide valuable information on the RBC distribution characteristics for relatively simple geometries.

Consequently, this study investigated RBC distribution characteristics using in vitro experiments with microfluidic channels of the order of 10 μm, which closely resemble capillaries, focusing primarily on ladder structures commonly observed near the termini of capillaries. The ladder structure model used in this study was of the anti-parallel type comprising six bifurcating channels, in which the flow directions at the inlet and outlet opposed one another. Using this model, the effects of variations in the parent channel width, distance between branches, and hematocrit on the RBC distribution in each bifurcating channel were evaluated. The findings provide valuable information regarding the physiological mechanisms of RBC phase separation in the microcirculatory system.

## 2. Materials and Methods

The microfluidic channels used in this study were fabricated using a standard soft lithography technique [34] with polydimethylsiloxane (PDMS). The channel was designed using DraftSight software (Dassault Systemes, Velizy-Villacoublay, France) and transferred onto a chrome photomask. A master mold was fabricated using photolithography by patterning SU-8 (10 μm thickness) (3010; KAYAKU Advanced Materials, Inc., Tokyo, Japan) on a silicon wafer. The PDMS prepolymer and curing agent (Sylgard 184 Silicone Elastomer Kit; Dow Corning Toray, Tokyo, Japan) were mixed in a 10:1 ratio and cast onto the SU-8 master to replicate the microchannel features. After curing, the PDMS replica was peeled from the SU-8 mold and bonded onto a glass slide after oxygen plasma treatment to complete the fabrication of the microchannels.

**Figure 1:**
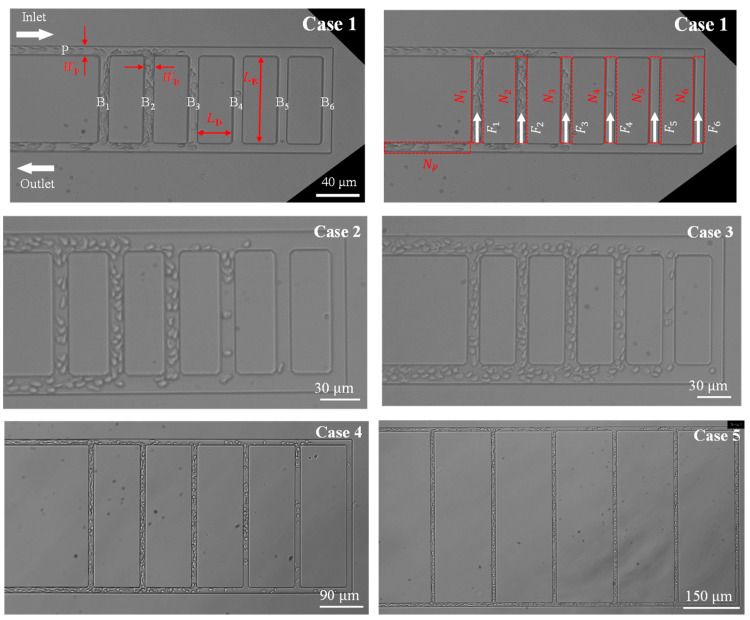
Geometries of microfluidic channels with the ladder structure used in the study, in which the flow directions at the inlet and outlet are reversed. The letter “P” denotes the parent channel, while “B_1_” to “B_6_” denote bifurcating channels 1 to 6. Five types of microfluidic channels were prepared by varying the parent channel width (*W*_P_), the distance between branches (*L*_D_) and the length of the bifurcating channels (*L*_B_). The bifurcating channel width (*W*_B_) was fixed in all cases. When comparing the parent channel widths, three different *W*_P_ values of 10, 13, and 16 µm were prepared, corresponding to Case 1, Case 2, and Case 3, respectively. When *L*_D_ varied, *W*_P_ and *W*_B_ were fixed at 10 µm, and *L*_D_ was varied to 90 µm, and 150 µm, respectively, designated as Case 4, and Case 5. Image analysis was conducted by considering the region of interest (ROI) indicated by the red dashed frames. $N_{P}$ and $N_{i}$ denote the number of RBCs within the ROI. $F_{i}$ denotes the number of RBCs passing through the bifurcating channel $B_{i}$.

In this study, we prepared an anti-parallel ladder structure model, as shown in Figure 1, in which the flow directions at the inlet and outlet were reversed. The letter “P” in the figure denotes the parent channel, while “B_1_” to “B_6_” denote bifurcating channels 1 to 6. Five types of microfluidic channels were prepared by varying the parent channel width (*W*_P_), the distance between branches (*L*_D_) and the length of the bifurcating channels (*L*_B_), as shown in Table 1. The bifurcating channel width (*W*_B_) was fixed in all cases. When comparing the parent channel widths, three different *W*_P_ values of 10, 13, and 16 µm were prepared, corresponding to Cases 1, 2, and 3, respectively. For each case, the dimensions of *L*_D_, *L*_B_, and *W*_B_ were fixed at 30, 90, and 10 µm, respectively. When *L*_D_ was varied, *W*_P_ and *W*_B_ were fixed at 10 µm, and *L*_D_ was varied to 90 µm and 150 µm, designated as Cases 4 and 5, respectively. *L*_B_ was set as *L*_B_ = 3·*L*_D_ to maintain the desired ratio. The channel height was set to 10 µm for all cases.

Human blood samples obtained from the Japanese Red Cross Society were centrifuged at 3000 rpm for 15 min to separate the blood components. The supernatant was removed, and the remaining solution was mixed with phosphate-buffered saline. This process was repeated several times to isolate the RBCs. Finally, the RBCs were suspended in phosphate-buffered saline containing bovine serum albumin (1 wt%) and dextran 40 (5 wt%). The experimental setup involved a syringe pump (Legato 111P, KD Scientific Inc., Holliston, MA, USA) attached to the outlet of the microchannel to control the flow rate to 1 m/s while withdrawing the RBC suspension. The RBC motion was observed using an inverted microscope (IX71; Olympus, Tokyo, Japan). All images were captured at 200 fps using a high-speed camera (K-II; Kato Koken, Isehara, Japan) and analyzed with DIPP-Motion V/2D fluid analysis software (Ditect Corporation, Tokyo, Japan) using particle tracking velocimetry.

The tube hematocrit values ($H_{p}$) for the parent channel P and ($H_{i}$) for the bifurcating channel $B_{i}$ could be obtained through image analysis by considering the region of interest (ROI) indicated by the red dashed frames in Figure 1. The tube hematocrit values were calculated as follows:

(1) $$H_{P}=\frac{N_{P}V_{\mathrm{RBC}}}{V_{\mathrm{ROI}}},\;H_{i}=\frac{N_{i}V_{\mathrm{RBC}}}{V_{\mathrm{ROI}}}$$

where $N_{P}$ and $N_{i}$ denote the number of RBCs within the ROI, $V_{\mathrm{RBC}}$ = 94.1 μm^3^ denotes the average volume of a single RBC, and $V_{\mathrm{ROI}}$ denotes the volume of the channel within the ROI. Subsequently, the fractional RBC flux in the bifurcating channel $B_{i}$ can be defined as $F_{i}$/$F_{P}$, where $F_{i}$ denotes the number of RBCs passing through the bifurcating channel $B_{i}$, and $F_{P}$ denotes the sum of $F_{i}$ for all bifurcating channels. In this study, three sets of RBC tracking processes were performed for each channel, with a duration of 1 s (300 frames) per set.

In the absence of RBC flow, the fractional flow ratio of the bifurcating channel $B_{i}$, $Q_{i}/Q_{P}$, can be theoretically determined. The flow rates of the parent channel P and bifurcating channel $B_{i}$ are denoted as $Q_{P}$ and $Q_{i}$, respectively. Based on Poiseuille’s law, the flow rate (*Q*) can be determined when a fluid flows through a cylindrical pipe of radius *r* and length *L*, as follows:

(2) $$Q=\frac{\pi r^{4}}{8\mu }\cdot \frac{\Delta p}{L}=\frac{\Delta p}{R}$$

where *μ* denotes the viscosity of the fluid, Δp denotes the pressure difference between the two ends of the cylindrical pipe, and $R=8\;\mu L/\pi r^{4}$ denotes the hydraulic resistance. Q, Δp, and R correspond to the current, voltage, and resistance, respectively, in Ohm’s law. Consequently, applying Kirchhoff’s law to the ladder structure allows the calculation of the fractional flow ratio $Q_{i}/Q_{P}$ in the bifurcating channel $B_{i}$. In this study, the hydraulic diameter was used because the cross-sectional microchannel was rectangular.

## 3. Results

**Figure 2:**
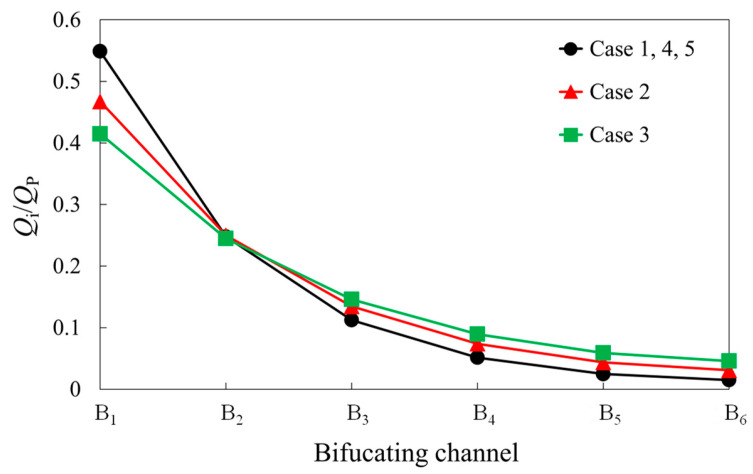
Theoretical fractional flow ratios into each bifurcating channel of the ladder structure model for five different cases.

We first investigated the theoretical fractional flow ratios in each bifurcating channel of the ladder structure in the absence of RBCs. Figure 2 shows a comparison of the fractional flow ratios in each bifurcating channel for the five cases. From the figure, it is evident that the fractional flow ratio into the bifurcating channels decreases rapidly as one moves away from the inlet. When comparing the different parent channel widths, as the width of the parent channel increases, the rate of decrease in the flow ratio becomes more gradual. However, when the ratio of the distance between the branches to the bifurcating channel length is the same (Cases 1, 2, and 3), the geometries are similar, resulting in identical fractional flow ratios in each bifurcating channel.

**Figure 3:**
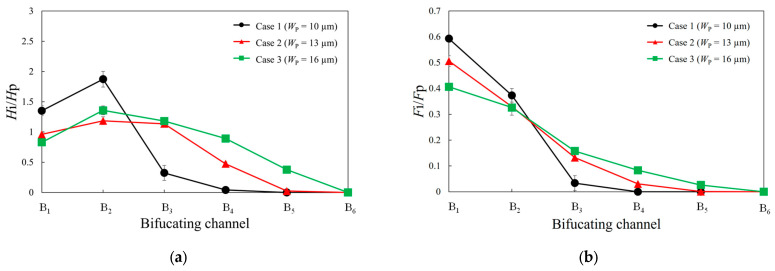
(**a**) Comparison of normalized hematocrit values and (**b**) fractional RBC flux for each bifurcating channel at three different parent channel widths.

Next, we present the experimental results for a suspension of RBCs flowing into a microfluidic channel. Figure 3a,b show a comparison of the hematocrit values and fractional RBC flux for each bifurcating channel with three different parent channel widths. Here, *L*_D_, *L*_B_, and *W*_B_ are fixed at 30, 90, and 10 µm, respectively. The average tube hematocrit in the parent channel is approximately 10%. Figure 3a shows that as the channel width decreases, the heterogeneity in the hematocrit distribution becomes more pronounced. In particular, for *W*_P_ = 10 μm, the hematocrit value of B_2_ surpasses that of B_1_. Figure 3b shows that as the width of the parent channel decreases, the bias in the fractional RBC flux increases.

**Figure 4:**
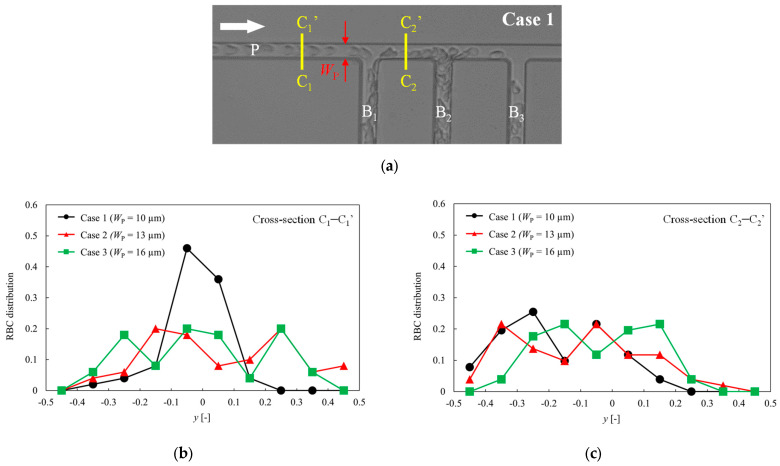
(**a**) Location of the measured cross-sections (C_1_–C_1_′ and C_2_–C_2_′). The cross-section C_1_–C_1_′ is located at the parent channel, and the cross-section C_2_–C_2_′ is located between the bifurcating channel B_1_ and B_2_. (**b**) Comparison of the RBC distributions at the channel cross-section C_1_–C_1_′ for different parent channel widths. (**c**) Comparison of the RBC distributions at the channel cross-section C_2_–C_2_′ for different parent channel widths.

To investigate the RBC flow characteristics before and after bifurcating channel B_1_ in more detail, a comparison of the RBC distributions in the channel cross section was conducted. As shown in Figure 4a, cross-section C_1_–C_1_′ is located at the parent channel, and cross-section C_2_–C_2_′ is located between the bifurcating channel B_1_ and B_2_. Figure 4b,c depict a comparison of RBC distributions at the channel cross-sections C_1_–C_1_′ and C_2_–C_2_′, respectively, where the horizontal axis is normalized by each channel width. In the case of *W*_P_ = 10 μm, the RBCs in the parent channel are aligned in a parachute shape, with a concentrated distribution near the channel center. By contrast, for *W*_P_ = 13 and 16 μm, the RBCs are uniformly distributed throughout the parent channel. From Figure 4c, it is evident that after passing through branch, the RBC distribution is biased toward the bifurcating channel B_1_ at channel cross-section C_2_–C_2_′ for *W*_P_ = 10 μm and 13 μm.

**Figure 5:**
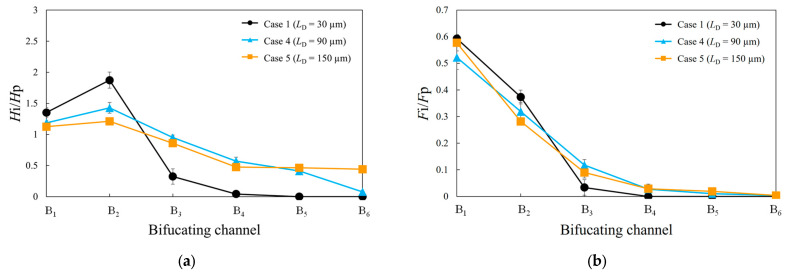
(**a**) Comparison of the normalized hematocrit values and (**b**) fractional RBC flux for each bifurcating channel at three different distances between branches.

Figure 5a compares the normalized hematocrit values for three different distances between the branches, the widths of the parent and bifurcating channels being fixed at 10 m. As shown in this figure, considerable heterogeneity in the hematocrit distribution among the bifurcating channels is evident for small *L*_D_. Additionally, the hematocrit value of channel B_2_ is higher than that of channel B_1_. Conversely, for *L*_D_ = 150 μm, the heterogeneity in the hematocrit distribution is reduced, and upon comparing the hematocrit values among the bifurcating channels, the maximum value is approximately twice the minimum value. Figure 5b presents a comparison of the fractional RBC flux for three different distances between the branches. The figure shows a rapid decrease in the fractional RBC flux with distance from the inlet. In particular, at *L*_D_ = 30 μm, the fractional RBC fluxes in channels B_4_ to B_6_ are almost zero.

**Figure 6:**
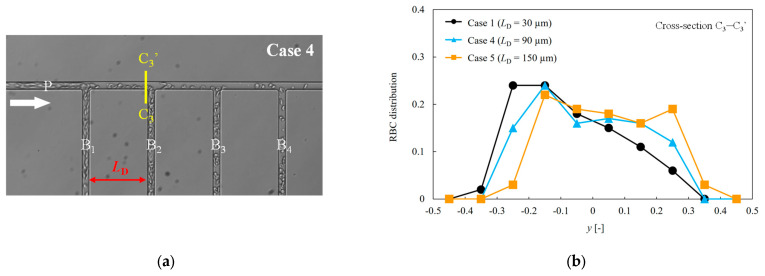
(**a**) Location of the measured cross-section (C_3_–C_3_′). The cross-section C3–C3 is located immediately before the bifurcating channel B_2_ (**b**) Comparison of the RBC distribution at channel cross-section C_3_–C_3_′ for different distances between branches.

We investigated the influence of differences in the distance between the bifurcating channels on the RBC flow within the channel. The RBC distribution at the channel cross-section (C_3_–C_3_′) immediately before the bifurcating channel B_2_ was analyzed, as shown in Figure 6a. Figure 6b presents a comparison of the RBC distribution at cross-section C_3_–C_3_′ for three different distances between branches (Cases 1, 4, and 5), where the horizontal axis is normalized by each channel width. From this figure, it is evident that in Case 1, the RBC distribution exhibits a bias toward the side of bifurcating channel B_2_. However, as the distance between branches increases, the bias in the RBC distribution diminishes, leading to a more symmetrical distribution.

**Figure 7:**
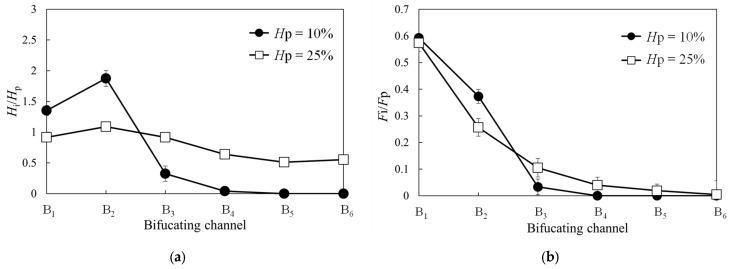
(**a**) Normalized hematocrit values and (**b**) fractional RBC fluxes for each bifurcating channel at parent channel tube hematocrits of 10% and 25%. Here, the channel geometry corresponds to Case 1.

Finally, we investigated the influence of hematocrit variations on the RBC distribution characteristics in bifurcating channels. Figure 7a,b compare the normalized hematocrit values and fractional RBC fluxes of each bifurcating channel at parent channel tube hematocrits of 10% and 25%, respectively. The channel geometry corresponds to that of Case 1. From the figure, it is evident that the hematocrit difference greatly affects the hematocrit distribution of the RBCs allocated to each bifurcation channel. Higher hematocrit levels mitigate the bias in the hematocrit distributions for each bifurcation channel. Furthermore, an increase in hematocrit slightly alleviates the pronounced heterogeneity in the fractional RBC fluxes.

## 4. Discussion

A capillary network exhibits a complex and intricate architecture that can be effectively evaluated by classifying it into its various components. Previous in vitro experiments have used honeycomb networks [15,16,17], square networks [17], and hierarchical networks [18]. In this study, we used soft lithography techniques to fabricate a microfluidic channel with multiple bifurcating channels of size approximately 10 μm, resembling a relatively simple ladder structure. We performed an image analysis to investigate the behavior of the RBCs distributed in each bifurcating channel. The experimental results demonstrated considerable variations in the hematocrit values and fractional RBC fluxes among the branching channels, depending on the width of the parent channel and the distance between the bifurcating channels. In particular, in Case 1, there was almost no RBC flow in bifurcating channels B_3_ to B_5_. This is undesirable in terms of the oxygen supply to the tissue. The heterogeneity of the RBC flux observed in this ladder structure has not been adequately evaluated quantitatively in previous studies. Therefore, this is one of the achievements that should be emphasized in this study. However, the relatively simple ladder geometry used in the study limits the information that can be obtained. Therefore, it is necessary to perform more in vitro experiments under more conditions in the future for a comprehensive understanding of RBC partitioning in the capillary network. In addition, although healthy RBCs were used in this study, the actual influx of pathological RBCs into the capillary network may alter RBC partitioning properties. For example, the low deformability of RBCs reduces their axial concentration and significantly changes the RBC distribution in the vessels. Therefore, it is important to investigate the impact of pathological RBCs on oxygen heterogeneity in future studies. The knowledge obtained from these investigations could aid in the study of diseases associated with impaired microcirculation.

We examined the hematocrit and fractional RBC flux of each bifurcating channel by varying the parent channel width from 10 to 16 μm while keeping the bifurcating channel width constant at 10 μm. The experimental results showed that as the parent channel width decreased, the heterogeneity in the RBC distribution among the bifurcating channels increased. This can be explained by comparing the RBC distribution in the cross section of the parent channel, as depicted in Figure 4. Figure 4a shows that for a smaller channel width, the erythrocyte flow in the parent channel assumes a parachute shape and tends to be more concentrated near the center of the channel. Consequently, as shown in Figure 4b, after passing through the branching point, the RBCs exhibit a greater bias toward the bifurcating channels. Therefore, for *W*_p_ = 10 μm, the majority of RBCs flowed into B_1_ and B_2_, whereas only a negligible amount reached B_3_ and beyond, resulting in a considerable bias in the RBC distribution in each bifurcating channel. Hyakutake et al. [18] reported that the RBC distribution before the bifurcation point has a major impact on the RBC distribution ratio after bifurcation. It can be inferred that a similar trend is evident in the present ladder structure.

We investigated the hematocrit and fractional RBC fluxes of each bifurcation channel by varying the distances between the bifurcating channels while maintaining a constant ratio of the bifurcating channel length to the bifurcating channel distance. Theoretical considerations suggest that in the absence of the RBCs, the fluid flow rate ratio in each bifurcating channel should be the same because of the constant ratio of the bifurcating channel length to the bifurcating channel distance. However, as shown in Figure 5, the fractional RBC fluxes of each bifurcating channel vary with the distance between the bifurcating channels. In particular, for *L*_B_ = 30 μm, the hematocrit of each bifurcation channel exhibits substantial differences compared to the other two cases. This can be explained by the RBC distribution in the channel cross section after bifurcating channel B_1_, as depicted in Figure 6. When the distance between bifurcating channels is short, the RBCs reach the next bifurcation without sufficient migration toward the channel center, resulting in a biased distribution that persists into the subsequent bifurcating channel. Consequently, when the distance between bifurcating channels is short, the heterogeneity between the bifurcating channels becomes more pronounced. However, as the distance between the bifurcating channels increases, the RBCs are transported to channels further away from the inlet. This phenomenon provides crucial insights for constructing a proper network geometry in the capillary network to efficiently supply oxygen to tissues.

We investigated the impact of hematocrit variations on the hematocrit and fractional RBC flux characteristics of bifurcating channels. We observed a substantial increase in the bias of the RBC distribution for each bifurcation channel as the hematocrit decreased. The hematocrit within the capillaries was considerably lower than that within normal blood vessels owing to the Fahraeus effect. Moreover, it is known to vary greatly depending on the microvascular network structure, with a typical range of 15–25%, exhibiting substantial variability. In this study, we examined the distribution characteristics of the RBCs by varying the tube hematocrit of the parent channel from 10% to 25%. The results revealed substantial differences in RBC distribution characteristics between the two hematocrit conditions. At a hematocrit of 10%, a small distribution of RBCs occurred toward the bifurcating channels B_4_ to B_6_. The distribution characteristics of RBCs toward the termini of capillaries are of utmost importance from an oxygen supply to tissues perspective. The findings of this study provide valuable insights into the optimal shape required to ensure efficient oxygen delivery to capillary termini when constructing microvascular network structures during angiogenesis.

## 5. Conclusions

In conclusion, this study focused on investigating the partitioning characteristics of RBCs within capillaries, with a specific emphasis on the ladder structures observed near the end of capillaries. In vitro experiments were conducted using microfluidic channels to evaluate the effects of various factors on RBC partitioning in bifurcating channels. The results revealed several key findings. First, as the parent channel width decreased, the heterogeneity in the hematocrit distribution and bias in the fractional RBC flux increased. This suggests that the width of the parent channel plays a major role in determining the RBC distribution within the capillaries. Second, variations in the distance between branches also affected the RBC distribution. Smaller distances between branches resulted in more significant heterogeneity in the RBC distribution. This finding highlights the importance of the geometric arrangement of capillary networks in influencing the RBC partitioning characteristics. Furthermore, the study demonstrated that the bias of the RBC distribution in the microchannel cross section significantly affected the RBC partitioning characteristics. The distribution of RBCs in the cross section of the microchannel was influenced by the width of the parent channel. An investigation into the influence of hematocrit variations on RBC distribution revealed that lower hematocrit values led to a more pronounced bias in RBC distribution. This indicates that changes in hematocrit levels can have a major impact on RBC partitioning within the capillaries.

Overall, this study provides valuable insights into the distribution characteristics of RBCs in capillary networks, contributing to a better understanding of the physiological mechanisms of RBC phase separation in the microcirculatory system. The knowledge gained from this study has implications for predicting oxygen heterogeneity in tissues and could aid in the study of diseases associated with impaired microcirculation, such as ischemic heart disease.

## Figures and Tables

**Table 1:** Parent channel width (*W*_P_), bifurcating channel width (*W*_B_), distance between branches (*L*_D_), and bifurcating channel length (*L*_B_) of each channel.

	*W*_p_ [μm]	*W*_B_ [μm]	*L*_D_ [μm]	*L*_B_ [μm]
Case 1	10	10	30	90
Case 2	13	10	30	90
Case 3	16	10	30	90
Case 4	10	10	90	270
Case 5	10	10	150	450

## Data Availability

Not applicable.

## References

[1] Goldsmith H.L. (1986). The microcirculatory society Eugene M. Landis award lecture: The microrheology of human blood. Microvasc. Res..

[2] Fung Y.C. (1997). Biomechanics: Circulation.

[3] Popel A.S., Johnson P.C. (2005). Microcirculation and hemorheology. Annu. Rev. Fluid Mech..

[4] Fung Y.C. (1973). Stochastic flow in capillary blood vessels. Microvasc. Res..

[5] Doyeux V., Podgorski T., Peponas S., Ismail M., Coupier G. (2011). Spheres in the vicinity of a bifurcation: Elucidating the Zweifach–Fung effect. J. Fluid Mech..

[6] Østergaard L., Kristiansen S.B., Angleys H., Frøkiær J., Michael Hasenkam J.M., Jespersen S.N., Bøtker H.E. (2014). The role of capillary transit time heterogeneity in myocardial oxygenation and ischemic heart disease. Basic Res. Cardiol..

[7] Pries A.R., Ley K., Claassen M., Gaehtgens P. (1989). Red cell distribution at microvascular bifurcations. Microvasc. Res..

[8] Pries A.R., Secomb T.W., Gaehtgens P., Gross J.F. (1990). Blood flow in microvascular networks. Experiments and simulation. Circ. Res..

[9] Clavica F., Homsy A., Jeandupeux L., Obrist D. (2016). Red blood cell phase separation in symmetric and asymmetric microchannel networks: Effect of capillary dilation and inflow velocity. Sci. Rep..

[10] Roman S., Merlo A., Duru P., Risso F., Lorthois S. (2016). Going beyond 20 μm-sized channels for studying red blood cell phase separation in microfluidic bifurcations. Biomicrofluidics.

[11] Shen Z., Coupier G., Kaoui B., Polack B., Harting J., Misbah C., Podgorski T. (2016). Inversion of hematocrit partition at microfluidic bifurcations. Microvasc. Res..

[12] Miyoshi Y., Abe H., Hyakutake T. In vitro evaluation of red blood cell flow in bifurcating microchannel. Proceedings of the IEEE 20th International Conference on Bioinformatics and Bioengineering.

[13] Stauber H., Waisman D., Korin N., Sznitman J. (2017). Red blood cell dynamics in biomimetic microfluidic networks of pulmonary alveolar capillaries. Biomicrofluidics.

[14] Kodama Y., Aoki H., Yamagata Y., Tsubota K. (2019). In vitro analysis of blood flow in a microvascular network with realistic geometry. J. Biomech..

[15] Mantegazza A., Clavica F., Obrist D. (2020). In vitro investigations of red blood cell phase separation in a complex microchannel network. Biomicrofluidics.

[16] Mantegazza A., Ungari M., Clavica F., Obrist D. (2020). Local vs. global blood flow modulation in artificial microvascular networks: Effects on red blood cell distribution and partitioning. Front. Physiol..

[17] Merlo A., Berg M., Duru P., Risso F., Davit Y., Lorthois S. (2022). A few upstream bifurcations drive the spatial distribution of red blood cells in model microfluidic networks. Soft Matter..

[18] Hyakutake T., Abe H., Miyoshi Y., Yasui M., Suzuki R., Tsurumaki S., Tsutsumi Y. (2022). In vitro study on the partitioning of red blood cells using a microchannel network. Microvasc. Res..

[19] Kihm A., Quint S., Laschke M.W., Menger M.D., John T., Kaestner L., Wagner C. (2021). Lingering dynamics in microvascular blood flow. Biophys. J..

[20] Pskowski A., Bagchi P., Zahn J.D. (2021). Investigation of red blood cell partitioning in an in vitro microvascular bifurcation. Artif. Organs..

[21] Rashidi Y., Simionato G., Zhou Q., John T., Kihm A., Bendaoud M., Krüger T., Bernabeu M.O., Kaestner L., Laschke M.W. (2023). Red blood cell lingering modulates hematocrit distribution in the microcirculation. Biophys. J..

[22] Li X., Popel A.S., Karniadakis G.E. (2012). Blood–plasma separation in y-shaped bifurcating microfluidic channels: A dissipative particle dynamics simulation study. Phys. Biol..

[23] Hyakutake T., Nagai S. (2015). Numerical simulation of red blood cell distributions in three-dimensional microvascular bifurcations. Microvasc. Res..

[24] Lykov K., Li X., Lei H., Pivkin I.V., Karniadakis G.E. (2015). Inflow/outflow boundary conditions for particle-based blood flow simulations: Application to arterial bifurcations and trees. PLoS Comp. Biol..

[25] Wang Z., Sui Y., Salsac A.-V., Barthès-Biesel D., Wang W. (2016). Motion of a spherical capsule in branched tube flow with finite inertia. J. Fluid Mech..

[26] Balogh P., Bagchi P. (2017). A computational approach to modeling cellular-scale blood flow in complex geometry. J. Comp. Phys..

[27] Balogh P., Bagchi P. (2017). Direct numerical simulation of cellular-scale blood flow in 3D microvascular networks. Biophys. J..

[28] Balogh P., Bagchi P. (2018). Analysis of red blood cell partitioning at bifurcations in simulated microvascular networks. Phys. Fluids.

[29] Ye T., Peng L., Li Y. (2018). Three-dimensional motion and deformation of a red blood cell in bifurcated microvessels. J. Appl. Phys..

[30] Ye T., Peng L., Li G. (2019). Red blood cell distribution in a microvascular network with successive bifurcations. Biomech. Model. Mechanobiol..

[31] Li G., Ye T., Li X. (2020). Parallel modeling of cell suspension flow in complex micro-networks with inflow/outflow boundary conditions. J. Comp. Phys..

[32] Li G., Ye T., Yang B., Wang S., Li X. (2023). Temporal-spatial heterogeneity of hematocrit in microvascular networks. Phys. Fluids.

[33] Ebrahimi S., Bagchi P. (2022). A computational study of red blood cell deformability effect on hemodynamic alteration in capillary vessel networks. Sci. Rep..

[34] Duffy D.C., McDonald J.C., Schueller O.J., Whitesides G.M. (1998). Rapid prototyping of microfluidic systems in poly(dimethylsiloxane). Anal. Chem..

